# Nonthionamide Drugs for the Treatment of Hyperthyroidism: From Present to Future

**DOI:** 10.1155/2018/5794054

**Published:** 2018-04-22

**Authors:** Nattakarn Suwansaksri, Lukana Preechasuk, Tada Kunavisarut

**Affiliations:** ^1^Department of Medicine, Khonkaen Hospital, Khon Kaen, Thailand; ^2^Siriraj Diabetes Center, Faculty of Medicine Siriraj Hospital, Mahidol University, Bangkok, Thailand; ^3^Division of Endocrine and Metabolism, Department of Medicine, Faculty of Medicine Siriraj Hospital, Mahidol University, Bangkok, Thailand

## Abstract

Hyperthyroidism is a common endocrine disease. Although thionamide antithyroid drugs are the cornerstone of hyperthyroidism treatment, some patients cannot tolerate this drug class because of its serious side effects including agranulocytosis, hepatotoxicity, and vasculitis. Therefore, nonthionamide antithyroid drugs (NTADs) still have an important role in controlling hyperthyroidism in clinical practice. Furthermore, some situations such as thyroid storm or preoperative preparation require a rapid decrease in thyroid hormone by combination treatment with multiple classes of antithyroid drugs. NTADs include iodine-containing compounds, lithium carbonate, perchlorate, glucocorticoid, and cholestyramine. In this narrative review, we summarize the mechanisms of action, indications, dosages, and side effects of currently used NTADs for the treatment of hyperthyroidism. In addition, we also describe the state-of-the-art in future drugs under development including rituximab, small-molecule ligands (SMLs), and monoclonal antibodies with a thyroid-stimulating hormone receptor (TSHR) antagonist effect.

## 1. Introduction

Graves' disease (GD) is the most common cause of hyperthyroidism in clinical practice [1]. While thionamide drugs are the mainstay of hyperthyroidism treatment and have high efficacy, some patients experience serious side effects such as agranulocytosis or hepatitis, which are contraindications for further thionamide usage [2]. Therefore, these patients usually need nonthionamide antithyroid drugs (NTADs) for control while waiting for definite treatments. Also, some situations that need rapid restoration of euthyroidism such as thyroid storm and preparation for emergency surgery usually require combination treatment with thionamide and NTADs [1, 3].

In this narrative review, we provide data about the mechanisms of action, indications, dosages, and side effects of NTADs that are currently used including iodine-containing compounds, lithium carbonate, perchlorate, glucocorticoids, and cholestyramine. Furthermore, we provide an up-to-date review of studies that have investigated drugs acting on the pathogenesis of GD including rituximab and treatment targeting the thyroid-stimulating hormone receptor (TSHR) as well as the future prospects for new therapies for GD that have not been mentioned together in previous reviews.

## 2. Current Therapies

**Figure 1:**
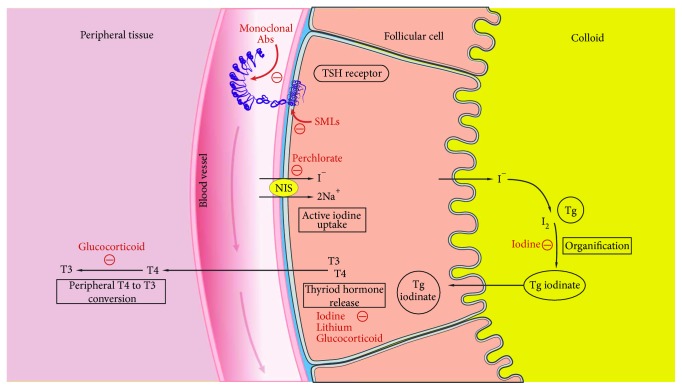
Mechanism of nonthionamide antithyroid drugs. Iodine-containing compounds mainly inhibit thyroid hormone release and transiently inhibit organification. Lithium also inhibits thyroid hormone release and may inhibit thyroid hormone synthesis. Perchlorate inhibits active iodide uptake by competitively binding with NIS. Glucocorticoid inhibits peripheral T4 to T3 conversion and may inhibit thyroid hormone secretion. MAbs act at the ectodomain of the TSH receptor while SMLs act at the transmembrane domain of the TSH receptor. MAbs: monoclonal antibodies; NIS: sodium iodide symporter; SMLs: small-molecule ligands; Tg: thyroglobulin; TSHR: thyroid-stimulating hormone receptor.

In this section, we describe currently available NTADs including their mechanisms of action, indications, and side effects. For quick reference, we have also summarized indications and dosing data in Table 1 and depicted the mechanisms of action in Figure 1.

### 2.1. Iodine-Containing Compounds

Iodine was used to treat hyperthyroidism before the discovery of thionamide antithyroid drugs. The iodine-containing compounds used in the treatment of hyperthyroidism are potassium iodide (KI) in the form of KI tablets, a saturated solution of potassium iodide (SSKI), and Lugol's solution. SSKI is prepared by adding KI crystals to water until the saturation point of KI is reached. Lugol's solution is an aqueous solution of elemental iodine and KI [4]. The concentration of iodide in each preparation might differ between manufacturers.

#### 2.1.1. Mechanisms of Action

The major actions of iodide on thyroid function are inhibition of thyroid hormone release from the thyroid gland and a transient decrease in thyroid hormone synthesis (the acute Wolff-Chaikoff effect) [4].

Previous studies have shown the putative mechanism of iodide's inhibition of thyroid hormone release from the thyroid gland. An *in vitro* study showed that excess iodide decreased thyroid hormone secretion by increasing the resistance of thyroglobulin to proteolytic degradation [5]. Wartofsky et al. demonstrated the onset, peak, and duration of iodide in hyperthyroidism. They administrated 120 mg of iodide (5 drops of Lugol's solution three times per day) to eight patients with hyperthyroidism and found that T4 secretion decreased as early as 12 hours after administration, reached a plateau effect within 3.5–6 days, and caused a sharp rise in serum T4 concentration to thyrotoxicosis range within 4 or 5 days after withdrawal of iodide [6].

Iodide causes a transient decrease in thyroid hormone synthesis. This mechanism is known as the Wolff-Chaikoff effect. It is an autoregulatory mechanism of the thyroid gland to handle excess iodine intake and prevent excessive thyroid hormone formation. In 1948, Wolff and Chaikoff showed that receiving a large amount of iodide stopped the organification of the thyroid cells in rats [7]. Nevertheless, the underlying mechanism of the acute Wolff-Chaikoff effect is still elusive. One proposed mechanism is an effect of the tri-iodine reaction that produces the tri-iodide anion, sequestering oxidized iodine and finally decreasing organification [8]. Other possible mechanisms are the inhibitory effect of high iodide concentration on thyroid peroxidase (TPO) function and the formation of organic iodocompounds called iodohexadecanal within the thyroid gland [9]. Because iodohexadecanal has multiple inhibitory effects on adenylate cyclase, NADPH oxidase, and TPO, it has been proposed to be the mediator of the Wolff-Chaikoff effect [10].

“Escape” from the acute Wolff-Chaikoff effect protects patients from hypothyroid state even though their high iodide status is continuous. If high iodide status is continuous, iodine transportation into the thyroid cell decreases because of the decreases in sodium iodide symporter (NIS) mRNA, NIS protein [11], and NIS uptake. After reducing intrathyroid iodine below the inhibitory level, thyroid iodination and thyroid synthesis resume. This is called “escape” from the acute Wolff-Chaikoff effect.

#### 2.1.2. Indication

*(1) Treatment of Thyroid Storm and Preoperative Preparation for Emergency Procedure*. High-dose iodine in combination with other drugs to treat thyroid storm can be used since iodide can quickly inhibit thyroid hormone release and thyroid hormone synthesis. The suggested dose of oral inorganic iodide for the treatment of thyroid storm is 200–2000 mg per day (Table 1) [3]. The American Thyroid Association (ATA) guideline [1] recommends that iodine be administered at least 1 hour after thionamide therapy to avoid further iodide organification [3]. However, the Japan Thyroid Association and the Japan Endocrine Society suggest that inorganic iodide be administered simultaneously with antithyroid drugs because they did not find any exacerbation of thyrotoxicosis in GD patients after treatment with methimazole (MMI) and KI in an outpatient Japanese population [12]. In thyroid storm treatment, many patients initially respond to iodine treatment, but this treatment loses its effect after 1-2 weeks [13, 14]. Therefore, iodine is usually prescribed for only 7–14 days [3].

The principles of preparation for emergency surgery are similar to thyroid storm treatment. These include rapid reduction of thyroid hormone level and control of hyperthyroidism symptoms with a combination drug regimen. Therefore, iodine-containing compounds at the same dose as thyroid storm treatment should be used in preoperative management and stopped immediately after surgery [3]. Furthermore, the ATA guideline suggests that most patients with GD should receive KI, SSKI, or Lugol's solution before thyroidectomy because of its beneficial effects including decreases in thyroid blood flow, vascularity, and intraoperative blood loss during thyroidectomy [1]. However, a recent extensive literature review found that the evidence on the use of preoperative Lugol's iodine for thyroidectomy in patients with GD was inconclusive due to weak evidence [15].

Some hyperthyroidism patients cannot receive iodine orally due to gastrointestinal problems. Alfadhli and Gianoukakis [16] proposed the proper management of severe thyrotoxicosis with gastrointestinal problems (Table 1).

*(2) Potential Treatment of Graves' Disease*. Although thionamide is the primary therapy in GD, some investigators have proposed a potential role for iodine monotherapy in drug-naïve patients with mild GD and patients with thionamide-associated side effects. In an observational study of drug-naïve patients, 20 mild GD patients receiving KI monotherapy (mostly KI 50 mg/d) were matched with 20 GD patients receiving MMI by propensity score analysis. This study demonstrated that iodine monotherapy could normalize thyroid function in 85% (17 of 20) of patients at 1 year after treatment. Ten percent of patients (2 of 20) initially responded to KI but showed recurrence of hyperthyroidism at 12–14 weeks after treatment. The rates of reduction of free T3, free T4, and thyrotropin receptor antibodies (TRAb) were similar between the KI and MMI groups [17].

In an observational study of GD with thionamide-associated side effects, 44 patients received KI 13–100 mg per day initially, and the doses were adjusted depending upon the biochemical responses. Sixty-six percent (29 of 44) of patients achieved normal thyroid function at a median of 35 days (range 8–329), and 38.6% of patients (17 of 44) were in remission after a median of 7.4 years (range 1.9–23) after KI therapy. After 32–609 days of treatment, 25% of patients (11 of 44) escaped from iodine responses [18]. Furthermore, prospective studies using combination treatment with both iodine and thionamide have shown that this combination could make patients euthyroid quicker than using thionamide drug alone, but this combination regimen did not improve the remission rate of GD [19, 20].

A previous retrospective study showed the benefit of KI as an adjunctive therapy of radioactive iodine (RAI) treatment. KI (around 250 mg/day) was administered at one week after RAI therapy and helped to shorten the duration of hyperthyroidism. However, patients taking KI more often developed transient hypothyroidism [21].

#### 2.1.3. Side Effects

Iodine has a few mild side effects such as rash, drug fever, sialoadenitis, conjunctivitis, mucositis, vasculitis, and leukemoid eosinophilic granulocytosis [22].

### 2.2. Lithium Carbonate

There are two lithium carbonate preparations, namely, immediate-release and sustained-release. The immediate-release and sustained-release preparations reach a peak plasma concentration at about 1-2 hours and 4-5 hours after administration, respectively. The elimination half-life of lithium is about 18–36 hours, and it is mostly excreted by the kidneys. Lithium clearance is considered to decrease with aging and renal impairment [23].

#### 2.2.1. Mechanisms of Action

Lithium is concentrated by the thyroid gland at a level 3 to 4 times of that in the plasma, probably by active transport [4]. The primary mechanism of lithium is the inhibition of thyroid hormone release [24] by inhibiting the action of TSH on cAMP [4, 25, 26]. Lithium may also inhibit thyroid hormone synthesis [4]. In hyperthyroidism patients, serum thyroxine mostly decreased to around 25–32% of baseline at 1 week after lithium treatment [27–29] and decreased to around 35% of baseline at 2 weeks after treatment [30]. However, responses to lithium treatment may vary. Ng et al. found that 8 of 13 thyrotoxicosis patients responded to lithium treatment (response defined as decreasing of free T4 > 50% with clinical improvement) as early as 2 weeks after treatment while 4 patients responded within 3 to 5 weeks after treatment (median dose of lithium 750 mg/d, range 500–1500 mg/d) [28].

It has been proposed that the thyroid gland may escape from the inhibitory effect of lithium. Nevertheless, data about this are sparse because most studies only used lithium for short durations. In 1974, lithium was used as monotherapy for 6 months in 11 patients with GD who relapsed from conventional therapy, and none of those patients escaped from the lithium effect [30] while Ng et al. found that only 1 of 13 patients escaped from the lithium effect after 12 weeks of treatment [28].

#### 2.2.2. Indication

*(1) Alternative Therapy for Hyperthyroidism*. Lithium is not the principal treatment of hyperthyroidism because of its side effects and a narrow therapeutic range. However, it can be used to temporarily control hyperthyroidism in patients who cannot use thionamide drugs. Lithium can also be prescribed as an alternative therapy in thyroid storm treatment [3]. The dose of lithium is 300–450 mg taken orally every 8 hours [4, 22]. Elderly patients require a lower dose of lithium to maintain the therapeutic level because they have a decrease in total body water and glomerular filtration rate. The recommended dose for elderly patients is shown in Table 1 [23, 31].

*(2) Adjuvant Treatment for Type 1 Amiodarone-Induced Thyrotoxicosis*. Lithium is not the first-line treatment of type 1 amiodarone-induced thyrotoxicosis (AIT) [32], but few studies have used lithium as adjuvant treatment. In a prospective study of 21 AIT patients, patients were nonrandomly allocated to 3 treatment groups including (1) amiodarone withdrawal group (*n* = 5), (2) propylthiouracil (PTU) group (*n* = 7), and (3) combination treatment of PTU (300 mg/d) and lithium (900–1350 mg/d) group (*n* = 9). Patients who received PTU had a normalized thyroid function at 11.6 ± 0.5 weeks while patients who received combination treatment of PTU and lithium had a normalized thyroid function at 4.3 ± 0.5 weeks [33]. One study demonstrated that administration of low-dose lithium in patients with AIT who did not respond to high-dose antithyroid drug combined with steroid could normalize thyroid function [34].

Lithium has been proposed as an adjunct to radioactive iodine (RAI) for the treatment of hyperthyroidism because lithium inhibits the release of iodine from the thyroid gland [35] and increases the retention of radioiodine without affecting thyroidal radioiodine uptake [36, 37]. Kessler et al. conducted a meta-analysis including 2 retrospective cohort studies, 3 randomized controlled trials, and a nonrandomized intervention trial. The retrospective cohort studies showed a significant improvement in the cure rate of hyperthyroidism at 1 year after RAI treatment in patients receiving RAI therapy with adjunctive lithium compared with those receiving RAI therapy only. The interventional trials showed an improvement in cure rate, but not statistically significant. The different results might be caused by a relatively low dose of RAI and shorter duration of antithyroid drug discontinuation before RAI in one study [38]. Adjunctive lithium reduced time-to-cure and blunted thyroid hormone excursion after RAI in both observational and interventional trials [38]. Most of the studies used lithium 900 mg/day divided into 3 doses [38], and the benefit of lithium was shown at lithium concentrations of 0.3 mEq/L or greater [39, 40]. The appropriate time of lithium initiation pre-RAI therapy and the duration of lithium treatment are inconclusive. Lithium should be prescribed before RAI therapy because lithium takes several days to reach peak serum level. Kessler et al. also suggested that the lithium duration of 10–14 days or possibly fewer is adequate for adjunctive treatment to RAI therapy [38]. However, there is still insufficient evidence to recommend adjuvant lithium to RAI therapy in clinical practice [1].

#### 2.2.3. Lithium Monitoring

Serum lithium should be monitored at 1 week after starting treatment as well as after dose adjustment and weekly until reaching the therapeutic level. The serum lithium concentrate should be maintained in the range of 1 mEq/L [4, 22]. A previous study showed that thyroid hormone secretion decreased rapidly after serum lithium concentrate reached 0.5 mEq/L [34]. Blood should be drawn for testing at 12 hours after the last dose [41].

#### 2.2.4. Side Effect

Symptoms of chronic intoxication can be classified to mild, moderate, and severe toxicity. The symptoms of mild toxicity (lithium level 1.5–2 mEq/L) include nausea, vomiting, diarrhea, hand tremor, and drowsiness while the symptoms of moderate toxicity (level 2–2.5 mEq/L) include myoclonic twitches, nystagmus, dysarthria, ataxia, and confusion. Severe toxic symptoms (level > 2.5 mEq/L) are renal impairment, impaired consciousness, seizure, coma, and death. Precipitating factors of chronic lithium toxicity might be an increase in the dose of the lithium regimen, a decline in renal function decline, or receival of some medications such as thiazides, nonsteroidal anti-inflammatory drugs, and angiotensin-converting enzyme inhibitors. These medications increase renal reabsorption of lithium, causing increased serum lithium concentration. However, toxic symptoms may occur even in the therapeutic range of lithium [42].

### 2.3. Perchlorate

Perchlorate is the dissociated anion of perchlorate salts. It is rapidly absorbed from the gastrointestinal tract after oral administration. Perchlorate reaches peak plasma concentration at 3 hours and has a half-life of approximately 6–8 hours. It is excreted by the kidney in an unchanged form [43, 44].

#### 2.3.1. Mechanisms of Action

Perchlorate inhibits iodide uptake in the thyroid gland by competitively binding with NIS and also has the ability to discharge iodine from the thyroid gland, reducing intrathyroidal iodine, thereby decreasing thyroid hormone synthesis [45]. Potassium perchlorate given orally resulted in a rapid release of accumulated intrathyroidal iodide in GD patients treated with thionamide [46].

#### 2.3.2. Indication

*(1) Adjuvant Treatment of Type 1 Amiodarone-Induced Thyrotoxicosis (Type 1 AIT)*. High-dose thionamide antithyroid drug is the drug of choice for type 1 AIT. However, the inhibitory effect of thionamide on thyroid hormone synthesis might be diminished in patients with type 1 AIT because of high iodine content in the thyroid cells. Therefore, perchlorate is a significant combination drug because it reduces thyroid iodine uptake. The recommended dose of potassium perchlorate is 1 gram per day or lower, divided into 2–4 doses per day for 2–6 weeks. Potassium perchlorate can be prescribed along with thionamide since the first diagnosis of type 1 AIT or as an adjuvant drug after failure to control hyperthyroidism by thionamide drug [32, 47, 48]. The questionnaire-based survey reported that European thyroidologists prefer to use thionamide and perchlorate in combination at the first diagnosis of type 1 AIT more than North American thyroidologists. The authors discussed that these differences might be explained from the difficulty in obtaining perchlorate in the United States and/or fears of its potential side effects [48].

#### 2.3.3. Side Effects

Perchlorate side effects are gastrointestinal irritation, rashes, drug fever, lymphadenopathy, nephrotic syndrome, and agranulocytosis. Furthermore, some cases of perchlorate-induced fatal aplastic anemia in hyperthyroidism treatment between 1961 and 1966 were reported in the literature. These patients received perchlorate doses ranging from 400–1000 mg per day. However, no cases of fatal aplastic anemia in patients receiving perchlorate for amiodarone-induced thyrotoxicosis have been reported [43].

### 2.4. Glucocorticoids

Previous studies have mostly used the oral form of dexamethasone to demonstrate the effects of glucocorticoid on the thyroid function test. However, both hydrocortisone and dexamethasone are commonly used as an adjuvant drug in the treatment of hyperthyroidism.

#### 2.4.1. Mechanisms of Action

The main mechanism of glucocorticoids in controlling thyrotoxicosis is their inhibitory effect on peripheral T4 to T3 conversion. Previous studies have demonstrated that the administration of dexamethasone 2 mg orally every 6 hours for 4 doses to patients with untreated GD decreased serum T3 by around 37–50%, and T3 began to decrease at 24 hours after the first dose of dexamethasone, subsequently decreasing persistently for at least 5 days [49, 50]. After dexamethasone administration, serum reverse T3 began to increase within 8 hours and increased by around 83%. This increase was caused by an increase in reverse T3 production without change in the clearance rate [50]. Those findings suggest that dexamethasone might increase type 3 deiodinase activity [51]. Furthermore, some investigators propose that dexamethasone might diminish thyroid hormone secretion in GD patients. The pieces of evidence supporting this mechanism are the reduction in T4 and thyroglobulin level after dexamethasone administration [49] as well as the persistent decrease in T3 level after the return of reverse T3 to baseline level [50].

#### 2.4.2. Indication

*(1) Treatment of Thyroid Storm or Preoperative Preparation for Emergency Procedure*. Glucocorticoids are used in combination therapy for thyroid storm treatment because of their inhibitory effect on the peripheral conversion of T4 to T3 and on thyroid secretion. Furthermore, there is some concern about relative adrenal insufficiency in hyperthyroidism [52, 53] and associated Addison's disease [54]. Hydrocortisone is usually given as 300 mg intravenous loading, followed by 100 mg every 8 hours [1], and dexamethasone 8 mg/day can be alternatively used in thyroid storm treatment [12]. The glucocorticoid dose should be tapered off if the signs of thyroid storm improve [1].

In emergency preoperative preparation for patients with hyperthyroidism, both hydrocortisone and dexamethasone can be used as adjuvant medication. The recommended doses are hydrocortisone 100 mg orally or intravenously every 8 hours or dexamethasone 2 mg orally or intravenously every 6 hours. The glucocorticoid dose should be tapered off in the first 72 hours after surgery [3].

#### 2.4.3. Side Effects

Glucocorticoids are usually prescribed in high doses and for short durations in the treatment of hyperthyroidism. Therefore, the side effects might include increased blood glucose, increased blood pressure, and suppression of immunological response [55].

### 2.5. Cholestyramine

Cholestyramine, an ion exchange resin, is a bile acid sequestrant. It decreases enterohepatic circulation of thyroid hormone and can be used to control hyperthyroidism.

#### 2.5.1. Mechanisms of Action

Both T3 and T4 are concentrated in the liver and secreted in the bile in conjugated form and a small amount in unconjugated form. Free hormones are released by bacterial enzyme deconjugation in the large intestine and reabsorbed to the blood circulation, completing the enterohepatic circulation of thyroid hormone [3, 56, 57]. Previous studies reported that cholestyramine decreased absorption of levothyroxine from the intestine [58, 59]. An *in vitro* study demonstrated that 50 mg of cholestyramine resin was capable of binding at least 3000 *μ*g of thyroxine, and the transportation of thyroxine across intestinal wall of rats was markedly inhibited by small amounts of cholestyramine [58].

#### 2.5.2. Indication

*(1) Alternative Therapy of Hyperthyroidism*. Cholestyramine might be prescribed as an alternative drug when the first-line therapy cannot be used for thyroid storm treatment [3]. Also, some case reports reported cholestyramine use in the treatment of contrast-induced hyperthyroidism in patients with multinodular goiters [60].

*(2) Adjuvant Therapy of Graves' Disease*. Previous studies have shown that combination therapy of cholestyramine and thionamide antithyroid drugs (MMI or PTU) can result in a euthyroid state in newly diagnosed GD patients more rapidly than thionamide antithyroid drugs alone [61–63]. The usual dose of cholestyramine is 4 grams orally two to four times a day. However, low-dose cholestyramine (1-2 grams orally two times a day) also demonstrated good efficacy [62]. Previous studies showed that the percent of reduction in free T4 at the end of the second and fourth week when combining thionamide and cholestyramine was 50–60% and 65–78%, respectively, and was 26–43% and 53–65%, respectively, in the MMI monotherapy group [61, 62]. There are no data about the long-term effect of cholestyramine in the treatment of GD because most studies used cholestyramine for only short durations (around 4 weeks). A combination of cholestyramine and thionamide antithyroid drugs might benefit patients with severe thyrotoxicosis symptoms requiring a rapid decrease in thyroid hormone.

#### 2.5.3. Side Effects

The common side effects of cholestyramine are bloating, flatulence, and constipation. However, most patients in previous studies could tolerate cholestyramine well. Since cholestyramine might bind to other drugs given concomitantly, it is generally recommended that other drugs be taken at least 1 hour before or 4 to 6 hours after cholestyramine [64].

## 3. Future Therapies

In the past few years, the mechanisms underlying GD have been elucidated in more detail [65, 66], and novel medications targeting the pathogenesis of GD have been widely investigated. These novel medications act mainly on B cell function and TSHR. However, the data of these new medications in humans are still limited.

### 3.1. Rituximab

B cells have many functions contributing to autoimmune thyroid disease including (1) being the precursor of plasma cells, which produce TRAb, a key feature of GD, (2) producing inflammatory cytokines, and (3) functioning as antigen-presenting cells [67].

Rituximab is a chimeric murine-human monoclonal antibody targeting CD20, which is a transmembrane protein expressed on pre, immature, mature, and memory B cells. Rituximab causes complete peripheral B cell depletion for at least 4–6 months by induction of apoptosis of B cells, antibody-dependent cellular cytotoxicity, and complement-mediated lysis [68]. Although most investigators have focused on the role of rituximab in the treatment of Graves' orbitopathy [67], some studies have investigated the effect of rituximab on thyroid function.

#### 3.1.1. Effect of Rituximab on Thyroid Hormone Level and Thyroid Autoantibodies

The effect of rituximab on thyroid hormone level in GD is inconclusive and is based on a few small phase 2 clinical trials [69, 70]. Only Heemstra et al. reported the benefit of rituximab in controlling hyperthyroidism [70]. In this prospective study, 13 relapsed GD patients received 2 doses of 1000 mg rituximab in 2-week intervals, and 9 patients responded to treatment with decreased free T4 and increased TSH and remained euthyroid for a median of 18 months (range 14–20 mo). The TRAb levels of 9 responders decreased significantly at 26 weeks after rituximab treatment. However, no correlation between TRAb level reduction and proportion of CD20 lymphocytes was found.

El Fassi et al. [69] conducted a small study of 20 newly diagnosed GD patients, and all patients were made euthyroid with MMI. Patients were nonrandomly allocated to a rituximab group and nonrituximab group equally. The result showed that patients in the rituximab group with low TRAb level had a higher rate of GD remission compared with the nonrituximab group at a median follow-up of 705 days (4 versus 0 patients, resp.). However, no difference in the rate of decrease in TRAb levels between groups was seen. Therefore, the authors concluded that rituximab might induce sustained remission of GD in patients with low TRAb levels.

#### 3.1.2. Reported Side Effects of Rituximab Therapy in Graves' Disease

All rituximab studies have reported some side effects with varying prevalence and severity. Before rituximab infusion, all patients were pretreated with acetaminophen, antihistamine, or glucocorticoid. The side effects included mild infusion-related adverse effects such as hypotension, nausea, fever, chill, and tachycardia as well as itching of the nose and throat. Other reported side effects were temporary joint pain, symmetric polyarthritis, serum sickness, and ulcerative colitis [69, 70].

### 3.2. Treatment Targeting the Thyroid-Stimulating Hormone Receptor

In GD, the TSHR is continuously stimulated by TRAb, causing an increased rate of thyroid hormone synthesis and secretion. TSHR is a G protein-coupled receptor comprising of three domains including a large amino-terminal ectodomain, a transmembrane domain and an intracellular carboxyl-terminal domain [71]. Researchers have been investigating treatments targeting the TSHR during the past few years. Two classes of these therapeutics are monoclonal antibodies (MAbs) to TSHR, which target the ectodomain of TSHR (orthosteric site), and small-molecule ligands (SMLs) that target the transmembrane domain of TSHR (allosteric site) [72]. An orthosteric site is defined as a classic binding site on a receptor, and an allosteric site is defined as a binding site that is topographically distinct from a classic orthosteric site. Because SMLs can be produced more easily and more cheaply compared to MAbs, many investigators have been developing therapeutic SMLs.

#### 3.2.1. Small-Molecule Ligands Binding to TSHR

SMLs can bind to a pocket within the transmembrane domain of TSHR, consequently inhibiting signaling that regulates the conformational changes of TSHR. SMLs do not affect the TRAb binding site at the ectodomain of TSHR. SMLs that might be developed for the treatment of GD are SML TSHR antagonists and SML TSHR inverse agonists.

NCGC00242595 (NIDDK-CEB-52) is an SML TSHR antagonist. In *in vitro* studies [73], this inhibited both activation of TSHR and upregulation of TPO expression by TSH and stimulated TRAb. NCG00242364 (ANTAG 3) was the first SML antagonist to demonstrate the antagonist effect of TSHR *in vivo* in a mouse model [74].

TSHRs are receptors that exhibit significant basal or constitutive signaling activity. Inverse agonists are ligands that inhibit receptor activation by agonists and additionally inhibit basal signaling [75]. To date, there have been three reported SMLs with inverse agonist activity including NCGC00161856, Org 274179-0, and NCGC00229600 [76]. All of these SMLs inverse agonists were studies *in vitro* and showed that they could inhibit basal and TSH-stimulated signaling in a model cell system [77, 78]. Furthermore, NCGC00229600 inhibited activation of TSHR signaling stimulated by TRAb from 30 sera of GD patients in a model cell system and a primary human thyrocyte culture [79]. Org 274179-0 has the highest potency compared with other SML antagonists or inverse agonists. However, it has the undesirable feature of being a partial agonist at the LH receptor [78].

Most of these SML TSHR still have a low potency that might not be clinically useful in GD treatment. Therefore, the development of more potent and more specific SML TSHR with antagonist activity is necessary [60].

#### 3.2.2. Thyrotropin Receptor Monoclonal Antibodies

TRAb are classified by their actions as stimulating, blocking, and neutral (or cleavage) antibodies [80]. Human MAbs to TSHR have been obtained from the peripheral lymphocytes of patients with autoimmune thyroid disease. Researchers have been developing blocking and inverse agonist MAbs for the treatment of immune and nonimmune hyperthyroidism in the future.

Until now, only two human MAbs have demonstrated TSHR antagonist effects. K1-70 is a MAb with TSHR antagonist activity, and 5C9 is a MAb with TSHR inverse agonist activity. In previous studies, both K1-70 and 5C9 could inhibit stimulation of TSH and TRAb from the serum of GD patients [81, 82], but only 5C9 could inhibit the basal activity of TSHR **[**81**]**. K1-70 was studied in rats with naturally high TSH and free T4 and could decrease the total and free T4 levels [83]. Furthermore, the safety, pharmacokinetics, and pharmacodynamics of K1-70 are being investigated in GD patients in a phase 1 clinical trial. MAbs with TSHR antagonist activity might be of benefit in other causes of hyperthyroidism such as TSH-secreting pituitary tumors [84] while SMLs and MAb inverse agonists of TSHR might clinically benefit patients with inherited nonimmune hyperthyroidism because of its ability to inhibit constitutive signaling of TSHR [71, 84]. Nonimmune hyperthyroidism is caused by germline mutation of TSHR, resulting in constitutive activation of the intracellular signaling cascade [22].

## 4. Conclusion

NTADs can be used to control symptoms of hyperthyroidism in some circumstances in which thionamide cannot be used or in combination with drug regimens. The mechanisms of action, indications, and side effects of NTADs are different. Understanding those differences can help the clinician select the appropriate drugs for the patients. Novel medications for GD such as rituximab, SMLs, and MAbs with TSHR antagonist effect are still being investigated.

## Figures and Tables

**Table 1:** Nonthionamide antithyroid drug dosage.

*Thyroid storm and preoperative preparation for emergent procedure*		
(1) Iodine-containing compounds (oral route)		
Iodide 200–2000 mg/d		[3]
Iodine-containing compound (in patients with gastrointestinal problem)		
SSKI 0.4 ml via sublingual every 8 h		[85]
SSKI 5–10 drops via rectal every 6–8 h		[16]
(2) Glucocorticoids		
(Thyroid storm)	Hydrocortisone 300 mg intravenous load then 100 mg every 8 h	[1]
	Dexamethasone 2 mg intravenously every 6 h	[12]
(Preoperative)	Hydrocortisone 100 mg orally or intravenously every 8 h	[3]
	Dexamethasone 2 mg orally or intravenously every 6 h	[3]

*Treatment of Graves' disease*		
(1) Iodine-containing compound (mild Graves' disease)		
KI 50 mg/d		[17]
(2) Cholestyramine (an adjuvant drug with a thionamide antithyroid drug)		
Cholestyramine 4 g orally every 6–12 h		[61, 63]
(3) Lithium carbonate		
Lithium 300 to 450 mg orally every 8 h		[4, 23, 31]
Age over 60 y: lithium 500 to 750 mg/d		
Age over 80 y: lithium should not exceed 450 mg/d		

*Type 1 amiodarone-induced thyrotoxicosis (as an adjuvant drug)*		
(1) Potassium perchlorate		
Potassium perchlorate 1 g/d (or lower) divided into 2–4 times/d		[32, 47]
(2) Lithium carbonate		
Lithium 300 to 450 mg orally every 8 h		[23, 31, 33]
Age over 60 y: lithium 500 to 750 mg/d		
Age over 80 y: lithium should not exceed 450 mg/d		

SSKI: saturated solution of 5% potassium iodide.

## Abbreviations

AIT: Amiodarone-induced thyrotoxicosis
GD: Graves' disease
KI: Potassium iodide
MAbs: Monoclonal antibodies
MMI: Methimazole
NIS: Sodium iodide symporter
NTADs: Nonthionamide antithyroid drugs
PTU: Propylthiouracil
SMLs: Small-molecule ligands
SSKI: Saturated solution of potassium iodide
TRAb: Thyrotropin receptor antibodies
TSHR: Thyroid-stimulating hormone receptor.
